# How Do Self-Esteem, Dispositional Hope, Crisis Self-Efficacy, Mattering, and Gender Differences Affect Teacher Resilience during COVID-19 School Closures?

**DOI:** 10.3390/ijerph19074150

**Published:** 2022-03-31

**Authors:** Ezza Mad Baguri, Samsilah Roslan, Siti Aishah Hassan, Steven Eric Krauss, Zeinab Zaremohzzabieh

**Affiliations:** 1 Department of Foundation Studies, Faculty of Educational Studies, Universiti Putra Malaysia, Serdang 43400, Malaysia; ezzamadbaguri2022@gmail.com (E.M.B.); z_zienab@upm.edu.my (Z.Z.); 2 Department of Counselor Education and Counseling Psychology, Faculty of Educational Studies, Universiti Putra Malaysia, Serdang 43400, Malaysia; siti_aishahh@upm.edu.my; 3 Department of Professional Development and Continuing Education, Faculty of Educational Studies, Universiti Putra Malaysia, Serdang 43400, Malaysia; lateef@upm.edu.my

**Keywords:** self-esteem, dispositional hope, mattering, crisis self-efficacy, teacher resilience

## Abstract

(1) Background: The closure of schools and the transition to online teaching because of the COVID-19 pandemic’s restrictions have resulted in significant changes in the workplace. Consequently, several resilience strategies have been implemented, and chief among them focus on the topic of burnout and coping abilities; (2) Purpose: Thus, this study investigates the influence of self-esteem, dispositional hope, and mattering on teacher resilience, and how crisis self-efficacy and gender differences mediate and moderate the relationships among associated variables. (3) Methods: This is a cross-sectional study with a cluster random sampling. A total of 248 secondary school teachers in Malaysia participated in this study. Questions were first transferred and formatted using a template of a commercial internet survey provider. Then, the university’s online learning platform was used both as a questionnaire distribution channel and a data collection method. Data analysis was conducted using structural equation modeling (SEM) with a partial least squares method; (4) Results: The findings of this study revealed that self-esteem, dispositional hope, and mattering significantly influence teacher resilience, and crisis self-efficacy mediates the impact of self-esteem and dispositional hope on teacher resilience. In some instances, the results also showed that gender has a moderating effect on teacher resilience during the pandemic; (5) Conclusions: This study used psychological factors to understand teacher resilience and incorporated crisis self-efficacy into teacher resilience research. It is one of the very few studies in resilience literature to investigate the moderating role of gender on teacher resilience.

## 1. Introduction

To halt the spread of the COVID-19 virus, many countries around the world resorted to school closures. Data published by UNESCO showed that school closures reached their peak in early April 2020, thus affecting approximately 1.6 billion students across 194 countries, which accounts for at least 90% of total student enrollment [1]. The sudden school closures forced teachers to find online and remote ways to support children’s learning and well-being in times of crises and disruption. Even in cases where schools were able to reopen, it has not been business as usual: teachers must deal with new health and safety regulations; many teachers are rightly concerned about the possible impact of COVID-19 on their health and safety; teachers have had to adapt their teaching to large groups of children who have missed out on learning and who struggle with socio-emotional well-being.

In response, many teachers had to reestablish workflow and adapt teaching practices in a short period, including having to hone technological and didactic skills and taking on the role of a learning manager. Accordingly, teachers identify primary concerns related to workload, a lack of support, and a lack of well-being or other psychological pressures to exit the profession. They express low job satisfaction with poor working conditions, thus implying burnout or coping struggles.

Burnout indicates that an individual has limited emotional and physical resources, that stressful work situations deplete these resources, and that when these resources are depleted, the individual, in this case, the teacher, is no longer able to function adequately [2,3]. Since burnout is an individual’s response, to remediate it, a person’s capacity to cope and adapt to challenging work needs to be fostered [4,5]. Thus, a call has been issued across a variety of occupational domains, including education, to increase employee resilience. Seaman and colleagues [6] defined resilience in public health as the ability of individuals to endure, adapt, and generate new ways of thinking and functioning in times of challenge, ambiguity, or hardship, allowing individuals to not only recover but, more importantly, thrive beyond crisis. The World Health Organization [7] also states that building resilience is a key factor in protecting and promoting health and well-being at both the individual and community levels.

Resilience has a number of positive effects for teacher education on a macro level, as well as for teachers and students on a micro-level. It specifically reduces job burnout and the mental health of teachers [8]. As a result, teachers must develop resilience to raise their tolerance for harsh working conditions. Improving teacher resilience is dependent on teachers’ coping techniques or strategies, as well as personal factors that may incline them to act as mediators [9]. Among the factors found in the literature, self-esteem [10], dispositional hope [11], mattering [12], and crisis self-efficacy [13] were found to play an important role in teacher resilience. According to Luthans et al. [14], the three components of psychological capital, self-efficacy, hope, and resiliency, as well as their interrelationships, enhance resistance to psychological distress. In a prior study, personal resources such as self-efficacy were found to be a mediator between work stress and job burnout among teachers [15].

As mentioned, self-esteem and mattering are two other personal factors that can influence teacher resilience. A previous study in Okinawa found a link between self-esteem and resiliency among teachers [16]. Richards et al. [17] also found perceived mattering is positively associated with resilience and inversely related to stress and burnout. In the same way, the effect of gender on individuals’ resilience is a matter of interest. Different studies point at how gender differences influence teachers’ core self-identities, namely personal and professional identities. Gender differences also play a role in adversity and resilience experiences lived by teachers [18,19]. Specifically, when teachers have a robust understanding of their own social identities, they tend to deal with adversity better.

To date, numerous studies have reported the link between self-reported resilience and positive outcomes, such as occupational well-being, psychosocial outcomes, commitment, and performance among teachers [11,20,21,22]. However, to address teacher stress and burnout amid COVID-19 through increased teacher resilience as advocated, it is important to adopt a critical and holistic approach with the caveat that not much is currently known about the contribution of self-esteem, dispositional hope, mattering, crisis self-efficacy, and gender to teacher resilience in the context of COVID-19 school closures. Thus, the first aim of this study was to examine the relationships between self-esteem, dispositional hope, mattering, and teacher resilience. The second aim of this study was to test the mediating role of crisis self-efficacy on the predictive relationships. In addition, this study aimed to seek the importance of gender differences in teacher resilience.

## 2. Literature Review

### 2.1. Self-Esteem and Teacher Resilience

One of the personality factors that potentially influence teacher resilience is self-esteem. Within the wider literature on resilience, self-esteem holds prominence in demonstrating that at high levels of it, one’s coping capacity towards adversity increases [23,24,25]. According to Mruk [26] and Jindal-Snape and Miller’s [27] self-esteem theory, self-esteem is defined as a personal attribute of persons who have survived in the face of difficult or risky conditions. The theory claimed that how people feel about themselves (self-esteem level) is influenced by their perceptions of themselves as valuable and accepted by others, as well as their judgments of competence in several domains. Previous studies have shown that the resilience and self-esteem connection was an important factor in teacher education, thus indicating a significant relationship [28,29]. However, there is a lack of studies that conducted in-depth analyses of this relationship among schoolteachers. Therefore, the current study proposed the following hypothesis:

**H1.**  *Self-esteem is positively related to teacher resilience.*

### 2.2. Dispositional Hope and Teacher Resilience

Derived from the theory of cognition, hope, as a dispositional construct, refers to a cognitive motivational factor that initiates and sustains goal-oriented actions [30]. The theory of hope [31] assumes that individuals are driven by goals. While they are motivated to attain and sustain positive goals, they tend to avoid or delay those that are negative. There are two elements of dispositional hope: (1) agency, which refers to the energy and motivation towards goal attainment, and (2) pathways, defined as the confidence to develop plans to achieve goals, especially in the presence of obstacles ahead [31,32]. Among relevant studies, hope has been linked to coping strategies that are active and approach-related [32] and a reduction in stress levels and depressive symptoms [33]. To date, no studies have examined the link between dispositional hope and resiliency among teachers, a cohort that experienced coronavirus school closures. Yet, the limited evidence indicates that hope was positively and significantly correlated with resilience [34]. Therefore, a second study hypothesis was proposed:

**H2.**  *Dispositional hope is positively related to teacher resilience.*

### 2.3. Mattering and Teacher Resilience

Perceived mattering refers to one’s self-acknowledgment that he/she is important to certain people’s lives [35]. Findings from notable studies reported that promoting mattering among teachers can effectively help them through challenging situations, such as the sweeping changes in schools caused by COVID-19. Considered as a psychological resource, mattering has been consistently associated with subdued depression in cross-sectional research, and it also serves as a barrier to depression which otherwise may develop over time [36,37,38,39]. Other studies on mattering at workplaces have found that mattering can be a stress-reducing factor at work [40] and workplace burnout [41]. To date, mattering has not been thoroughly explored or experimentally tested as a resilience factor; thus, a third study hypothesis was proposed:

**H3.**  *Mattering is positively related to teacher resilience.*

### 2.4. Crisis Self-Efficacy as a Mediator

Crisis self-efficacy refers to the belief that task completion is possible amid crises [42]. Park and Avery [43] characterized crisis self-efficacy as action efficacy, preventive efficacy, achievement efficacy, and uncertainty management efficacy. Accordingly, when situated in a crisis, Schwarzer and Warner [44] stated that an individual with high self-efficacy beliefs can have a positive impact on motivational processes despite the absence of specific stressors. However, being self-efficacious may also be helpful to show resilience in the face of adversity. Benight and Cieslak [45] also support the mediating effect of self-efficacy on personality factors and health outcomes. In addition, Hong et al. [46] found that self-efficacy significantly reduces depression and improves the psychological well-being of the Lebanese during the COVID-19 crises. It also played the mediating role of anxiety and psychological well-being of this population in the same crisis. However, no studies have been conducted to investigate crisis self-efficacy as a mediator of teacher resilience. Highly resilient teachers were found to be more capable of dealing with stress [47]. Efficacy is also associated with a teacher’s belief that they are effective as educators [48]. Individuals with strong self-efficacy can handle a task for a longer period than those with low self-efficacy, according to research. As a result, resilience and self-efficacy are also interrelated [46,48]. Accordingly, the fourth and fifth study hypotheses were proposed:

**H4.**  *Crisis self-efficacy is positively related to teacher resilience.*

**H5.**  *Crisis self-efficacy mediates the relationship between the predictors and teacher resilience.*

### 2.5. Gender as a Moderator

Gender differences is a factor of interest. In the stress-related literature, gender has been studied to understand how influential it can be in managing challenging life events. Many studies reported distinct male–female signatures towards resiliency. Thus, resilience has been viewed as an end-goal that can be achieved via numerous distinct and sometimes surprising paths, where males and females employ different mechanisms [49]. In a recent study of 1065 Israeli adults in the context of the pandemic-resultant lockdown, gender differences were examined in the areas of psychiatric symptomatology (depression, anxiety, and somatization), coping strategies, levels of resilience, and belief in a just world (BJW) [50]. It was found that women employed more coping tactics that focused on both emotions and problems than did men. However, men displayed higher resiliency and BJW compared to women. Yet, no known studies have explored gender as a moderator of teacher resilience. Therefore, a sixth study hypothesis was proposed:

**H6.**  *Gender moderates the relationship between the predictors and teacher resilience.*

## 3. Methodology

### 3.1. Research Design, Sampling, and Data Collection

A cross-sectional study was carried out to investigate the correlation among variables. The target population of the quantitative study was secondary school teachers in Malaysia. According to the Ministry of Health (MOH), throughout the pandemic outbreak, all states in Malaysia had recorded several numbers of confirmed COVID-19 cases from education clusters [51]. Therefore, the schools’ management were directed to ensure that teachers conduct teaching and learning via online platforms. In addition, the sample of respondents was selected at two stages of cluster random sampling. Using the fishbowl technique, one District Education Office (PPD) was randomly chosen out of the total PPDs of each state. This technique provides equal chances for the accessible population to be assigned as the sample.

In the next stage, respondents were selected within the cluster group. In each PPD, classes from five schools were randomly selected using the fishbowl technique again, so that each respondent in the population had an equal chance and probability of being selected without exception. Overall, a total of 248 secondary teachers in Malaysia were selected for this research. Before the surveys were distributed via the online platform, permission to distribute was sought and granted by the MOE, State Education Departments (JPNs), PPDs, and school principals of the selected schools. The link to the survey was shared with the school principals and/or management and blasted to the teachers. Respondents consist of 191 female teachers (77%) and 57 male teachers (23%), out of which 108 (62%) teach at primary schools while 66 (38%) teach at secondary schools. As a self-administered survey, teachers were asked to state their gender according to their own volition; therefore, gender was self-stated by the respondents. The average age of respondents is 41.65 (SD = 10.07), ranging from 22 to 62. Years of teaching experience average at 15.50 (SD = 9.88), ranging from 1 to 40 years.

### 3.2. Measures

*Resilience*: The brief resilience scale (which includes three positive and three negative items) [52] was used to evaluate one’s ability to recover or bounce back from a stressful situation. Examples of items include: “*I do not take a long time to recover from stress*” and “*I can bounce back after hardship*”. The Cronbach’s alpha for this measure was 0.75.

*Crisis self-efficacy*: Developed by Park and Avery [43], this scale consists of 13 items that assess four interrelated components of action, preventive, achievement, and uncertainty management. The original instrument showed a range of construct reliability from 0.85 to 0.92. Examples of items include: “*I can use resources effectively during a crisis,*” and “*During a crisis, I can stick to my goals*”.

*Mattering*: Amundson [53,54] developed this scale of six items (which includes three positive and three negative items) that assesses the four dimensions of attention, importance, dependence, and ego-extension. The Cronbach’s alpha for this measure was 0.918.

*Dispositional hope*: With seven items, the Adult Dispositional Hope Scale [32] assesses respondents’ perceived motivations for pursuing their goals (agency thought) and their ability to identify workable routes to goal attainment (pathway thought). The original instrument showed a range of construct reliability from 0.74 to 0.84. Examples of items include: “*I feel pretty successful in life*” and “*I will most of the time pursue my goals energetically*”.

*Self-esteem*: Using six items, Rosenberg’s [55] self-esteem scale allows respondents to indicate the extent to which they felt they possessed good qualities, accepted their characteristics, achieved personal success, or experienced failure. The Cronbach’s alpha for this measure was 0.87.

Items are rated on a 5-point Likert scale, with 1 indicating “strongly disagree” and 5 indicating “strongly agree.”

### 3.3. Data Analysis

To test the study hypotheses, Partial Least Squares Structural Equation Modeling (PLS-SEM) was used [56]. Specifically, the SmartPLS 3.3.7 [56] was used to perform this analysis. In line with Anderson and Gerbing’s [57] and Chin’s [58] two-step approach, first, our measures were validated, and then our hypothesized model was tested using the SmartPLS 3.3.7 software. The generation of standard errors and t values of the parameters were made via bootstrapping of 5000 samples. Before employing the SmartPLS 3.3.7 software (Oststeinbek, Germany) for analyses, we used the Statistical Package for the Social Sciences (SPSS version 26 New York, NY, USA) software to estimate the amount of missing data and found that the rate of missing data for items was less than 2%. Subsequently, we used the regression imputation method to address the missing data.

## 4. Analysis and Findings

### 4.1. Descriptive Statistics

Table 1 presents the means, standard deviation, and inter-correlation for the study variables. The results of this study show a value ranging from 0.422 to 0.719, thus indicating the correlation between all study variables as positive and significant. These correlation coefficient values are considered good indicators to proceed the analysis to the next stage.

### 4.2. Measurement Model Assessment

The PLS-SEM was used to compute a reflective measurement and structural model in this study [59]. The indicators and construct reliability (CR), convergent, and discriminate validity were tested in the reflective measurement model [60]. Table 2 shows that items with loadings less than 0.70 were removed from further analysis, since each item’s loading has to be greater than 0.50 [61], and the CR of all constructs surpassed 0.70 [59], indicating that the constructs were reliable.

As illustrated in Table 2, the average variance extracted (AVE) of the constructs was greater than 0.5 [59]. To examine discriminating validity, Fornell and Larcker’s [62] criteria were combined with heterotrait–monotrait (HTMT) ratios of relations [63]. We uncovered that discriminant validity was achieved because the square root of the AVE of each construct was greater than the correlation values of any construct pairs, according to the Fornell–Larcker criteria (Table 3), and also because the HTMT standards were all less than the 0.85 cutoff value (Table 4). As a result, this study demonstrated that dispositional hope, mattering, self-esteem, crisis efficacy, and resiliency can be distinguished.

### 4.3. Structural Model Assessment

**Figure 1:**
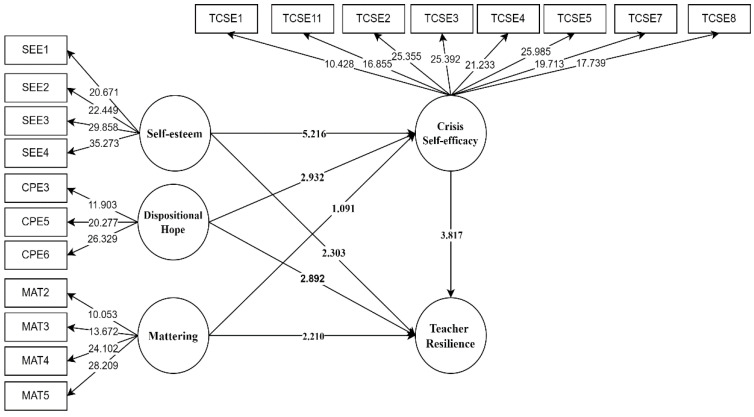
Diagram for the structural model of the study.

The bootstrapping approach was then used to assess the structural model (see Table 5). The findings indicate that self-esteem (β = 0.204, *p* = 0.019, f^2^ = 0. 0.04), dispositional hope (β = 0.199, *p* = 0.005, f^2^ = 0.039), mattering (β = 0.151, *p* = 0.016, f^2^ = 0.026), and crisis self-efficacy (β = 0.305, *p* = 0.000, f^2^ = 0.118) all exhibit positive and significant associations with teacher resilience. Thus H_1_−H_4_ were supported (Table 5 and Figure 1).

The influence of crisis self-efficacy as a mediator was then investigated. Table 5 shows the dispositional hope → crisis self-efficacy → teacher resilience (β = 0.063, *p* = 0.041, BC 0.95% LL = 0.021 and UL = 0.143) and self-esteem → crisis self-efficacy → teacher resilience (β = 0.115, *p* = 0.006, BC 0.95% LL = 0.051 and UL = 0.212) were both significant. Furthermore, the indirect effects did not straddle a 0 in between, demonstrating mediation, as suggested by Preacher and Hayes [64]. As a result, the mediation effect can be considered to be statistically significant, indicating that H_5a_ and H_5c_ were also supported (Table 5). However, the mattering → crisis self-efficacy → teacher resilience was not significant (β = 0.029, *p* = 0.356, BC 0.95% LL = −0.026 and UL = 0.098) and H_5b_ was rejected. In crisis self-efficacy and teacher resilience, the predicted variables explained 34.8 percent and 49.1 percent of the variation, respectively. The blindfolding approach was also employed to test the model’s predictive relevance. All of the Q^2^ values in this investigation were more than zero, with teacher resilience Q^2^ = 0.280 and crisis self-efficacy Q^2^ = 0.181 [65]. These figures indicate that the model is sufficiently predictive.

### 4.4. Moderation Effect of Gender

To investigate the moderating role of gender, the data were separated into male and female groups and measured independently in the modified model. The multi-group analysis was undertaken to confirm the moderating role of gender using the partial least squares multi-group analysis (PLS-MGA) [66]. Table 6 shows the results of the multi-group analysis. In terms of dispositional hope (0.344 versus 0.093, *t* = 2.085, *p* < 0.05), male teachers had a higher level of resilience than female teachers. In the case of self-esteem (−0.008 versus 0.448, *t* = 2.803, *p* < 0.001), females scored a higher level of resilience than their male counterparts. Thus, H_6a_ and H_6b_ were supported. However, the values for mattering (0.118 versus 0.065, *t* = 0.350, *p* > 0.05) and crisis self-efficacy (0.368 versus 0.231, *t* = 0.873, *p* > 0.05) were not significant at 5% level of significance. While mattering and crisis self-efficacy were not significantly moderated by gender, gender differences significantly moderated the effects of dispositional hope and self-esteem on teacher resilience.

## 5. Discussion and Implications

The COVID-19 pandemic put online teaching and learning to the test, which induced the acceleration of the digitalization of school teaching. This study attempts to fill a research gap of investigating predictors of teacher resilience in the said context. While many studies focused on teachers’ stress, burnout, and general psychological well-being during the pandemic-restriction context [67] in various countries [68], to the best of our knowledge, this study is the first to propose that self-esteem, dispositional hope, mattering, and crisis self-efficacy are valuable direct and indirect predictors of teacher resilience. This study also provides an account of the cumulative knowledge of how the impact of psychological factors on teacher resilience was affected by the moderating role of gender. Finally, this study was the first attempt to identify the influence of psychological factors on teacher resilience in the Malaysian context. Structural equation modeling with a partial least squares method was conducted to test the hypotheses. The findings of this study indicated that self-esteem, dispositional hope, and mattering explained 40.1% and 34.8% of the variance in crisis self-efficacy and teacher resilience, respectively.

Self-esteem is the first individual factor that was assumed to be a positive predictor of teacher resilience. The results of this study confirmed this hypothesis. This finding is consistent with previous research of Kurniawan et al. [69] that found self-esteem as a significant positive predictor of resilience. It means that high self-esteem levels would have a connection with teacher resilience in the COVID-19 crisis. The framework of Terror Management Theory (TMT) can explain this relationship; when individuals experience surging distress, they tend to boost their self-esteem and self-perceived value so that their psychological health can be preserved [70].

Dispositional hope also significantly influenced the teacher resilience, analogous with Yıldırım and Arslan’s [34] cross-section study which reported the correlation between dispositional hope and adult resilience at the onset of COVID-19. Therefore, teachers who keep their hopes high display higher recovery ability in highly stressful situations, and this facilitates increased resilience. Teachers with high dispositional hope are highly motivated with an ability to plan alternatives in light of challenges in teaching remotely during COVID-19 restrictions. These teachers have a deep belief that they are in control and can overcome the situation and difficulties. Holding such beliefs can lead to improved psychological health in teachers. Yıldırım and Arslan [34] suggested that it is important to develop online training programs that focus on hope and resilience to cater to those who have minimal contact with people. These programs can ensure the psychological health of those vulnerable to the risk of COVID-19 restrictions and lockdown.

Moreover, the impact of mattering on teacher resilience was significant, and the results are consistent with past research [71]. Extant evidence indicates that if mattering levels are low, the individual is more vulnerable to elevated depression levels [72]. Flett et al.’s [73] exploration on the mattering–perfectionism–depression link confirmed the correlation of higher depressive symptoms, reduced mattering, and increased levels of trait perfectionism and perfectionistic self-presentation. Observations reported that volunteering in meaningful ways fosters mattering [74], so ample volunteer opportunities should be created for teachers, especially among those who have few social connections and work remotely during the COVID-19 pandemic.

Furthermore, this study showed that crisis self-efficacy was a significant predictor of teacher resilience, which is consistent with the previous research of Yada et al. [75] and Kim and Burić [76]. The finding of this study indicates that teachers’ crisis self-efficacy drives them towards resilience. Teachers’ high efficacy belief in effectively managing unforeseen circumstances at work caused by the pandemic is a predictor of their high resilience to fulfill their professional and pedagogical functions and responsibilities in school. Chun [77] suggested that agility and adaptability training should be provided to teachers to face unanticipated work changes. Additionally, schools can develop more effective message strategies to enhance teachers’ crisis self-efficacy as a means to safeguard teachers, parents, students, and stakeholders from the risks of COVID-19 in the educational system.

The analysis also revealed that crisis self-efficacy mediated the effects of self-esteem and dispositional hope on teacher resilience. These results support past research that showed a positive association between self-esteem, dispositional hope, and resilience, as well as self-efficacy playing the mediator role [78]. In the recent development of the positive appraisal style theory of resilience (PASTOR), self-efficacy has been recognized as an important mechanism to attain resilience [79,80]. The findings also confirm that teachers with high self-esteem and hope are more likely to have high self-efficacy in difficult situations and eventually achieve resilience. It is observed that teachers’ self-belief in ability significantly influences their capability to not only maintain psychological and physical well-being but also to adapt to the challenges imposed by COVID-19.

The analysis also revealed that teacher resilience was gender-dependent and confirmed the moderating role of gender in teacher resilience. In terms of dispositional hope, female teachers had a higher resilience level than male teachers. Surprisingly, the results contradict previous findings [81,82]. These studies found no significant gender differences in the relationship between hope and resilience. This is because, according to Braun-Lewensohn et al. [81], female teachers are very important and influential in communal and household spheres in collectivist societies such as Malaysia. As a result, in times of stress or crisis, the responsibility they bear obligates them to act optimally, and as a result, they do not expose themselves to vulnerability. However, in terms of self-esteem these findings revealed that male teachers were more resilient. This conclusion is reinforced by previous meta-analytic evidence that men consistently outperform women in terms of self-esteem [83].

## 6. Limitations and Future Research Directions

The first limitation of the current study is the inclusion of a self-reported data collection method and the use of the cross-sectional method. Therefore, future studies are recommended to employ the longitudinal method and other methods to collect data, including interviews and observations. Secondly, this study could not conduct a variance analysis for gender in the proposed model because the sample sizes between the male and female cohorts were unequal. It is recommended that future studies perform a variance analysis for gender on the proposed model by considering the equal approximates of sample sizes of both genders. The third limitation of this study was that it was conducted on a non-clinical sample that produced results indicating the vulnerability of teachers to burnout. Therefore, future studies could attempt to reproduce the present results in a clinical sample (e.g., teachers with a clinical burnout diagnosis). Finally, the study was conducted in a South Asian country. Although the data support the study relationships, future research may focus on Western countries to enhance the generalizability of the finding.

## 7. Conclusions

The findings of this study revealed that to raise the resilience of teachers working during the COVID-19 pandemic, their self-esteem, hope, and mattering need to be improved. The results also indicate that crisis self-efficacy mediates the mentioned variables (i.e., self-esteem and hope) and teacher resilience. In addition, this research is expected to help us understand the role of gender in teacher resilience. It is found that male teachers had a higher teacher resilience level for dispositional hope than female teachers. In contrast, for self-esteem, female teachers had a higher level of teacher resilience than male teachers. Therefore, special attention should be paid to cultivating and developing teachers’ psychological attributes, particularly self-esteem, to enhance their resilience. With the findings of this study, it is hoped that all stakeholders will double up their efforts to improve and maintain the resiliency level of teachers who are coping with work challenges imposed by the COVID-19 crisis.

## Figures and Tables

**Table 1:** Mean, standard deviation, and inter-correlation among variables.

	Mean	SD	1	2	3	4
1. Dispositional hope	5.06	0.55	1			
2. Resilience	5.06	0.79	0.719 **	1		
3. Self-esteem	5.52	0.74	0.650 **	0.551 **	1	
4. Mattering	5.17	0.67	0.588 **	0.487 **	0.524 **	1
5. Crisis self-efficacy	5.04	0.69	0.631 **	0.591 **	0.519 **	0.422 **

Note. ** Correlation is significant at the 0.01 level (2-tailed).

**Table 2:** Measurement model assessment.

Constructs	Items	Loading	α	rho_A	CR	AVE
HOP			0.68	0.707	0.82	0.604
	CPE3	0.701				
	CPE5	0.816				
	CPE6	0.81				
MAT			0.752	0.764	0.843	0.576
	MAT2	0.751				
	MAT3	0.744				
	MAT4	0.807				
	MAT5	0.822				
RES			0.848	0.85	0.892	0.623
	RES1	0.804				
	RES2	0.779				
	RES3	0.83				
	RES4	0.812				
	RES6	0.715				
SEE			0.791	0.796	0.864	0.614
	SEE1	0.759				
	SEE2	0.754				
	SEE3	0.794				
	SEE4	0.826				
CSE			0.887	0.891	0.91	0.559
	CSE1	0.728				
	CSE11	0.704				
	CSE2	0.769				
	CSE3	0.771				
	CSE4	0.783				
	CSE5	0.777				
	CSE7	0.734				
	CSE8	0.709				

Note. Dispositional Hope = HOP, Mattering = MAT, Resilience = RES, Self-esteem = SEE, Crisis self-efficacy = CSE.

**Table 3:** Fornell–Larcker criterion.

	1	2	3	4	5
1. HOP	0.777				
2. CSE	0.5	0.747			
3. MAT	0.596	0.435	0.759		
4. RES	0.57	0.585	0.521	0.789	
5. SEE	0.629	0.561	0.578	0.588	0.784

Note. Dispositional Hope = HOP, Mattering = MAT, Resilience = RES, Self-esteem = SEE, Crisis self-efficacy = CSE.

**Table 4:** HTMT criterion.

	1	2	3	4
1. HOP				
2. CSE	0.622			
3. MAT	0.808	0.521		
4. RES	0.71	0.666	0.651	
5. SEE	0.835	0.66	0.739	0.71

Note. Dispositional Hope = HOP, Mattering = MAT, Resilience = RES, Self-esteem = SEE, Crisis self-efficacy = CSE.

**Table 5:** Hypotheses testing.

Hypotheses	Relationships	Std β	*t*-Value	*p*-Value	BC 0.95% LL	BC 0.95% UL	Decision
H_1_	HOP → RES	0.199	2.892	0.005	0.046	0.332	SU
H_2_	CSE → RES	0.305	3.817	0.000	0.156	0.477	SU
H_3_	MAT → RES	0.151	2.210	0.016	0.032	0.269	SU
H_4_	SEE → RES	0.204	2.303	0.019	0.028	0.362	SU
H_5a_	HOP → CSE →RES	0.063	2.044	0.041	0.021	0.143	SU
H_5b_	MAT →CSE → RES	0.029	0.923	0.356	−0.026	0.098	NS
H_5c_	SEE →CSE → RES	0.115	2.752	0.006	0.051	0.212	SU

Note. Dispositional Hope = HOP, Mattering = MAT, Resilience = RES, Self-esteem = SEE, Crisis self-efficacy = CSE, Supported = SU, Not supported = NS.

**Table 6:** Partial least squares results for the moderating effects of gender.

Hypotheses	Relationships	Male		Female		*t*-Value Difference	*p* Values	Decision
		Std β	*t*-Value	Std β	*t*-Value			
H_6a_	HOP → RES	0.344	3.792	0.093	1.156	2.085	0.039	SU
H_6b_	SEE → RES	−0.008	0.074	0.448	3.757	2.803	0.006	SU
H_6c_	MAT → RES	0.118	1.164	0.065	0.572	0.350	0.727	NS
H_6d_	CSE → RES	0.368	3.792	0.231	2.189	0.873	0.384	NS

Note. Dispositional Hope = HOP, Mattering = MAT, Resilience = RES, Self-esteem = SEE, Crisis self-efficacy = CSE, Supported = SU, Not supported = NS.

## Data Availability

The data presented in this study are available on request from the corresponding author.
